# Combined inhibition of BCL-2 and MCL-1 overcomes BAX deficiency-mediated resistance of *TP53*-mutant acute myeloid leukemia to individual BH3 mimetics

**DOI:** 10.1038/s41408-023-00830-w

**Published:** 2023-04-24

**Authors:** Bing Z. Carter, Po Yee Mak, Wenjing Tao, Edward Ayoub, Lauren B. Ostermann, Xuelin Huang, Sanam Loghavi, Steffen Boettcher, Yuki Nishida, Vivian Ruvolo, Paul E. Hughes, Phuong K. Morrow, Torsten Haferlach, Steven Kornblau, Muharrem Muftuoglu, Michael Andreeff

**Affiliations:** 1 Section of Molecular Hematology and Therapy, Department of Leukemia, The University of Texas MD Anderson Cancer Center, Houston, TX USA; 2 Department of Biostatistics, The University of Texas MD Anderson Cancer Center, Houston, TX USA; 3 Department of Hematopathology, The University of Texas MD Anderson Cancer Center, Houston, TX USA; 4 Department of Medical Oncology and Hematology, University Hospital Zurich and University of Zurich, Zurich, Switzerland; 5 Oncology Research, Amgen Inc, Thousand Oaks, CA USA; 6 Amgen Inc, Thousand Oaks, CA USA; 7 MLL Munich Leukemia Laboratory, Munich, Germany

**Keywords:** Cancer, Leukaemia

## Abstract

*TP53*-mutant acute myeloid leukemia (AML) respond poorly to currently available treatments, including venetoclax-based drug combinations and pose a major therapeutic challenge. Analyses of RNA sequencing and reverse phase protein array datasets revealed significantly lower BAX RNA and protein levels in *TP53*-mutant compared to *TP53–*wild-type (WT) AML, a finding confirmed in isogenic CRISPR-generated *TP53*-knockout and -mutant AML. The response to either BCL-2 (venetoclax) or MCL-1 (AMG176) inhibition was BAX-dependent and much reduced in *TP53*-mutant compared to *TP53*-WT cells, while the combination of two BH3 mimetics effectively activated BAX, circumventing survival mechanisms in cells treated with either BH3 mimetic, and synergistically induced cell death in *TP53*-mutant AML and stem/progenitor cells. The BH3 mimetic–driven stress response and cell death patterns after dual inhibition were largely independent of *TP53* status and affected by apoptosis induction. Co-targeting, but not individual targeting of BCL-2 and MCL-1 in mice xenografted with *TP53*-WT and *TP53*-R248W Molm13 cells suppressed both *TP53*-WT and *TP53*-mutant cell growth and significantly prolonged survival. Our results demonstrate that co-targeting BCL-2 and MCL-1 overcomes BAX deficiency-mediated resistance to individual BH3 mimetics in *TP53*-mutant cells, thus shifting cell fate from survival to death in *TP53*-deficient and -mutant AML. This concept warrants clinical evaluation.

## Introduction

Patients with *TP53*-mutant acute myeloid leukemia (AML) respond poorly to current therapies. Consequently, *TP53* mutations are frequently enriched/selected in refractory/relapsed AML [1–4], and associated with low survival rates [5–8].

The combination of the BCL-2 inhibitor venetoclax (VEN) with a hypomethylating agent (HMA) or low-dose Ara-C elicits complete remission/complete remission with incomplete hematologic recovery rates of >65%, is well tolerated by elderly AML patients [9, 10], and has been approved by the US Food and Drug Administration. This combination has also shown promising results in refractory/relapsed AML [11]. Apoptosis induction depends highly on intact p53 signaling [12]. Not surprisingly, patients with *TP53* mutations have vastly inferior responses to VEN-based therapies [13–16], supported by a recent pre-clinical study demonstrating that *TP53* mutations confer resistance to HMA/VEN in vitro and in vivo [17] and CRISPR genome editing analyses demonstrating that the loss of p53-regulated apoptosis networks confers resistance of AML cells to VEN [18]. Furthermore, a retrospective analysis showed that adding VEN to standard therapies does not improve the outcomes of patients with *TP53*-mutant AML [19].

Mitochondria-mediated apoptosis is controlled by pro- and anti-apoptotic BCL-2 proteins. We and others have reported that MCL-1, a major resistance factor for BCL-2 inhibition, is frequently induced in VEN-resistant AML cells [20–22] and that co-inhibition of BCL-2 and MCL-1 is highly synergistic, both in vitro and in vivo, against various malignant cells, including AML cells and stem/progenitor cells, that have intrinsic or acquired resistance to VEN [18, 23–28]. In addition, *BAX* mutations can confer VEN resistance to AML cells [29]. We reported that combined p53 activation and BCL-2 inhibition is synthetically lethal in *TP53*-WT AML by promoting pro-apoptotic BCL-2 proteins and targeting MCL-1 [21]. We, therefore, postulated that directly targeting MCL-1 may partially compensate for p53 deficiency and enhance VEN activity in *TP53*-mutant AML.

Alterations in the balance of pro- and anti-apoptotic BCL-2 proteins could restrict the response of *TP53*-mutant cells to BH3 mimetics. We determined RNA and protein levels of BCL-2 family members in large cohorts of *TP53*-mutant AML and investigated if shifting the balance of BCL-2 proteins to pro-death by co-targeting BCL-2 and MCL-1 could overcome *TP53*-mutant AML resistance to individual BH3 mimetics. We here report that decreased expression of BAX in *TP53*-defective AML cells contributes to their resistance to BH3 mimetics. Co-targeting of BCL-2 and MCL-1 overcomes BAX deficiency-mediated resistance of *TP53*-mutant AML and AML stem/progenitor cells to individual BH3 mimetics in vitro and in xenografts comprising both *TP53*-WT and -mutant Molm13 cells in vivo.

## Materials and methods

### Cells, cell culture, and treatment

Molm13 cells were purchased from the German Collection of Microorganisms and Cell Cultures (Braunschweig, Germany). U937, KG1, and THP1 cells were purchased from ATCC (Manassas, VA). Cell lines were validated by STR DNA fingerprinting using the AmpF_STR Identifier kit according to the manufacturer’s instructions (Applied Biosystems). The STR profiles were compared to known ATCC fingerprints, and to the Cell Line Integrated Molecular Authentication database (CLIMA) version 0.1.200808 (http://bioinformatics.istge.it/clima/) [30]. The STR profiles matched known DNA fingerprints or were identified as unique. Mycoplasma was tested with Detection Kit from Applied Biological Materials (Richmond, BC, Canada) per the manufacturer’s instructions. *TP53-*R248W Molm13 [31] and *TP53*-knockout (KO) and -mutant (R175H, Y220C, M237I, R248Q, R273H, and R282W) Molm13 cells [32] were generated as described previously. BAX-knockdown (KD) and vector control Molm13 cells [33] and p53-KD and control OCI-AML3 cells [34] were generated as described previously. Primary AML peripheral blood samples with *TP53* mutations were obtained from patients (Table 1) after obtaining written informed consent following protocols approved by MD Anderson Cancer Center Institutional Review Board and in accordance with the Declaration of Helsinki. Human bone marrow (BM)-derived mesenchymal stromal cells (MSCs) were isolated from normal donors as described previously [35]. Cell lines and mononuclear cells from primary samples were cultured under previously described conditions [28]. AML cell lines without and primary cells with MSC co-cultures (4:1 ratio) were treated with DMSO, VEN, AMG176, or the combination.

**Table 1:** Patient characteristics.

Patient no.	Blast %	*TP53* mutations (VAF%)	Other mutations	Previous treatments and responses
26	50	c.659 A > G p.Y220C (15)	*NF1, PTPN11, NRAS, KRAS*	Resistance to rituximab, palbociclib, and fulvestrant
		c.427 G > A p.V143M (14)		
28	95	c.733 G > A p.G245S (87)	*NF1, TET2, DNMT3A*	Resistance/relapsed AML. Resistance to chemotherapy and to **VEN**-based therapy
29	89	c.403 T > G p.C135G (86)	*NF1, DNMT3A*	Resistance/relapsed AML. Resistance to azacitidine/**VEN**, and BP1001 + decitabine
30	85	c.994-1 G > A (70)	*NF1, ASXL1, CEBPA, KDM6A, RUNX1*	Resistance/relapsed AML. Resistance to 7 + 3, fludarabine- idarubicin-**VEN**, PLX51107 + azacitidine, daratumumab + **VEN**
32	88	c.742 C > T p.R248W (80)		Resistance to cladribine and low-dose cytarabine, azacitidine, **VEN** and pevonedistat, PLX51107 + azacitidine.
33	65	c.517 G > A p.V173M (46)		Resistance to azacitidine
		c.376-1 G > A (43)		
34	94	c.814 G > A p.V272M (22)	*ASXL1, CEBPA*	Newly diagnosed
		c.537 T > A p.H179Q (25)		
35	78	c.445dup p.S149fs (89)	*BCOR, TET2, IDH2, STAG2, DNMT3A*	Refractory AML. Resistance to fludarabine, cytarabine, filgrastim, idarubicin, **VEN**, 5-azacitidine plus **VEN**, LY3410738.

### Generation of VEN and AMG176–resistant cells

To generate AML cells with acquired resistance to both BCL-2 and MCL-1 inhibitors, we treated MV4-11 cells with acquired resistance to VEN previously generated [21] with increasing concentrations of AMG176 over a period of ~2 months.

### RNA-seq analysis

We performed differential gene expression analysis between *TP53*-mutant (*n* = 63) and -WT (*n* = 668) AML patient samples collected at diagnosis using a dataset from Dr. Torsten Haferlach, MLL Munich Leukemia Laboratory, and the Torsten Haferlach Leukemia Diagnostic Foundation, Munich, Germany. These sample sizes are sufficient to provide 99% power for a 0.5 log fold change, with the false discovery rate being controlled under 5%.

### Reverse phase protein array

Proteomic profiling of >200 proteins in samples obtained from newly diagnosed AML patients were performed by reverse phase protein arrays (RPPA) following the custom-built AML array [36–38]. Among these samples, 122 were from de novo AML patients with known *TP53* gene status, who had not been exposed to chemotherapy. The log2-normalized expression levels of BCL-2 family proteins in this cohort were analyzed and compared between the *TP53*-WT (*n* = 101) and -mutant (*n* = 21) samples. These sample sizes are sufficient to provide 90% power for a 0.5 log fold change, with the false discovery rate being controlled under 5%.

### Cell viability assay

Viable cell numbers and apoptosis were determined by flow cytometry as previously described [28]. For primary samples co-cultured with MSCs, Annexin positivity was determined in bulk (CD45^+^), and leukemia stem/progenitor cells (CD34^+^) and expressed as specific apoptosis [22, 39]:

$$\frac{{{{{\mathrm{\% }}}}\;{{{\mathrm{of}}}}\;{{{\mathrm{apoptosis}}}}\;{{{\mathrm{in}}}}\;{{{\mathrm{treated}}}}\;{{{\mathrm{cells}}}} - {{{\mathrm{\% }}}}\;{{{\mathrm{of}}}}\;{{{\mathrm{apoptosis}}}}\;{{{\mathrm{in}}}}\;{{{\mathrm{untreated}}}}\;{{{\mathrm{cells}}}}}}{{{{{\mathrm{\% }}}}\;{{{\mathrm{of}}}}\;{{{\mathrm{viable}}}}\;{{{\mathrm{untreated}}}}\;{{{\mathrm{cells}}}}}} \times 100{{{\mathrm{\% }}}}$$

### Western blot

Western blot was carried out and analyzed as described previously [27] using the Odyssey Infrared Imaging System for signal detection and Odyssey software version 3.0 for quantification (LI-COR Biosciences; Lincoln, NE). Antibodies against BCL-2 (#M088729-2) were purchased from Dako/Agilent (Santa Clara, CA); against BCL-2A1 (#14093), BCL-XL (#2764), MCL-1 (#94296), and BID (#8762), from Cell Signaling Technology (Danvers, MA); against BAX (#B8554), from Sigma; against BIM (#ab32158), NOXA (#ab13654), and PUMA (#ab186917), from Abcam (Cambridge, MA); against BAK (#NBP1-74026), from Novus Biologicals (Centennial, CO); and against p53 (DO-1) (#sc-126), from Santa Cruz Biotechnology, Inc. (Dallas, TX). β-actin was used as a loading control.

### BAX and BAK activation assay

Activation of BAX and BAK was determined following the described assay [40]. Antibodies against BAX (clone 6A7) (#556467) and BAK (#556382) were purchased from BD Biosciences, San Diego, CA. Cytochrome *C* (#612310) (BioLegend, San Diego, CA) release was determined concomitantly to assess loss in mitochondrial membrane potential.

### Multi-modality cell death assay

Cell fates were determined using a multi-metric cell death assay [41], modified from a previously described assay [42]. Briefly, cells were stained with a live/dead cell dye (Tonbo Biosciences, San Diego, CA); fixed and permeabilized; stained with antibodies against various cell death and survival markers, including ATF4 (#10835-1-AP) (Proteintech, IL), p21 (#5487 S) (Cell Signaling Technology), RIP3 (#sc-374639) (Santa Cruz Biotechnology, Inc.), γ-H2AX (#613420) (BioLegend), Ki-67 (#563756) (BD Biosciences), LC3B (#8899) (Cell Signaling Technology), and cleaved-caspase 3 (#560627) and cleaved-PARP (#564130) (both from BD Biosciences); and subjected to flow cytometric analysis. The percentages of dead cells and cleaved-caspase 3 or cleaved-PARP positive cells among all cells were calculated, and the geometric means (G-means) of ATF4, p21, RIP3, γ-H2AX, Ki-67, and LC3B in the live cells were determined. Data were expressed as UMAP plots and heatmaps. Prior to performing high-dimensional analysis, we excluded debris, gated on singlets, and exported events, including both live and dead cells, for downstream analysis. Pooled cells from all the experimental conditions were subjected to UMAP dimension reduction and FlowSOM clustering (Omiq.ai). Arcsinh-transformed or scaled median signal intensities were used to generate heatmaps or data plots in R software version 3.6.3 (R Foundation for Statistical Computing, Vienna, Austria; https://www.R-project.org) as described previously [43]. The Seurat package [44] was used to cluster *TP53*-WT, -deficient, and -mutant (R175H and R248Q) leukemia cells treated with DMSO, VEN, AMG176, or both based on the expression levels of the features assessed using flow cytometry. UMAP embedding based on 4 principal components was used to separate different experimental conditions.

### In vivo experiments

Mouse experiments were conducted in accordance with MD Anderson’s Institutional Animal Care and Use Committee-approved protocols. Molm13 cells (0.30 × 10^6^ luciferase/GFP-labeled *TP53*-WT plus 0.03 × 10^6^ BFP-labeled *TP53-*R248W cells) were injected into the tail veins of 6- to 8-week-old male NSG mice (strain-005557, The Jackson Laboratory, Sacramento, CA). Once engraftment was confirmed by luciferase imaging with the IVIS-200 noninvasive bioluminescence in vivo imaging system (Xenogen, Hopkinton, MA), mice (5 or 6/group) were treated with vehicle (25% hydroxypropyl-β-cyclodextrin, pH 9), VEN (50 mg/kg, 6-day on/1-day off), AMG176 (30 mg/kg, day-2 and day-3 of each week), or VEN and AMG176 combination. Both agents were provided by Amgen and given via oral gavage. Disease progression and treatment responses were monitored with luciferase imaging and flow cytometric measurement of circulating GFP- and BFP-positive cells on day-3 of each week. Mouse survival was followed.

### Statistical analyses

Cell line experiments were performed in independent triplicates; results were expressed as means ± SEMs. The Student *t*-test was used to assess differences between groups. All tests are two-sided and *P* values ≤0.05 were considered statistically significant. Multiple testing was adjusted by the Benjamini–Hochberg procedure so that the resulting false discovery rate would be ≤0.05. These analyses were performed using the publicly available software package limma (https://bioconductor.org/packages/release/bioc/html/limma.html). The combination index (CI) [45], determined using Calcusyn software, was expressed as mean CI values at ED_50_, ED_75_, and ED_90_. A CI <1 indicates synergistic; CI = 1, additive; and CI >1, antagonistic effects. The Kaplan–Meier method was used to estimate mouse survival, and survival data were analyzed using the log-rank test.

## Results

### BAX expression is significantly decreased in *TP53*-mutant AML cells

**Fig. 1:**
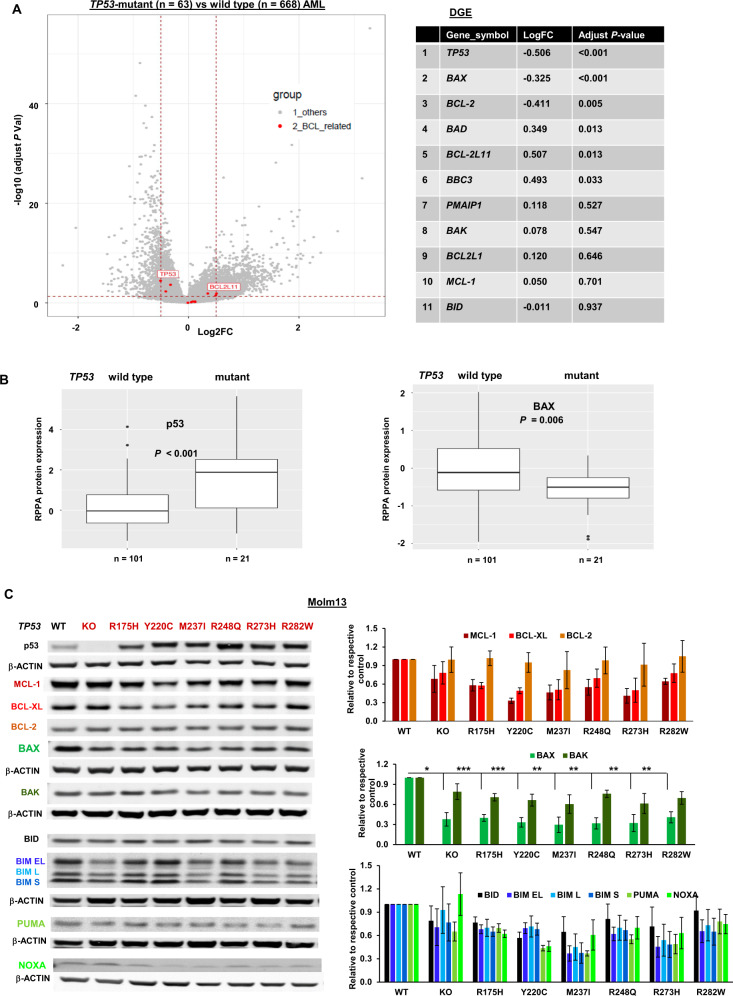
BAX expression is significantly decreased in *TP53*-KO and -mutant AML cells. **A** Volcano plot showing differential gene expression (DGE) and the adjusted *P* values of *BCL-2* family members between *TP53*-mutant and *TP53-*WT AML cells, as assessed by RNA-seq analysis. DGE and adjusted *P* values were calculated using the limma package. *TP53* and differentially expressed *BCL-2*–related genes are highlighted in red. **B** RPPA analysis of the log2-normalized expression levels of p53 and BAX in newly diagnosed de novo *TP53*-mutant and *-*WT AML. Box is 75 to 25% and bar in the middle is the median of expression. The whiskers are 95% CI, with the extremes (black dots) outside that. **C** Expression of BCL-2 family proteins in *TP53*-KO and -mutant Molm13 cells and isogenic WT control Molm13 cells. Left, a representative Western blot; right, the relative protein expression levels quantified from three Western blots. Error bars represent the mean ± SEM of three independent experiments. Statistical analysis was performed using a Student *t*-test, and *P* < 0.05 was considered statistically significant. *P < 0.05; **P < 0.01; ***P < 0.001.

To understand the potential mechanisms underlying resistance to VEN-based therapies in *TP53*-mutant AML, we first assessed the expression of BCL-2 proteins in AML. RNA-seq analysis compared the expression of *BCL-2* family members in *TP53*-mutant (*n* = 63; VAF = 4.46 to 99.2%; distribution shown in supplemental Fig. 1) with *TP-53* WT (*n* = 668) samples at diagnosis. The volcano plot, generated using the ggplot2 package in R, has a *P* cutoff of 0.5 Log2 fold change (FC) and 0.05 of adjusted *P* value cutoff (Fig. 1A). Highlighted are *TP53* and the differentially expressed *BCL-2*–related genes. Although none of the *BCL-2* members made the *P* cutoff of 0.5 Log2FC, significant decreases in *BAX* and *BCL-2* and increases in *BCL-2L11* (BIM), *BAD*, and *BBC3* (PUMA) were observed (adjust *P* < 0.05) (Fig. 1A). We next analyzed the levels of various BCL-2 proteins using an RPPA dataset generated from a large cohort of newly diagnosed de novo AML patients with known *TP53* genotype status (*n* = 122). As expected, p53 protein levels were significantly higher in *TP53*-mutant (*n* = 21) than in *TP53-*WT AML (*n* = 101; *P* < 0.001). *TP53* VAFs were not available and could vary greatly among these samples. Even so, we found that only the BAX levels were significantly lower in *TP53-*mutant than in *TP53-*WT AML (*P* = 0.006) among BCL-2 family proteins (Fig. 1B and Supplemental Fig. 2).

To validate that *TP53*-deficiency is indeed responsible for BAX reduction, we determined the expression of BCL-2 proteins in isogenic Molm13 cells with *TP53*-WT, *TP53*-KO, or various *TP53* hotspot mutations [32]. Compared with WT controls, *TP53-*defective cells show variably altered expression of various BCL-2 proteins. Among them, BAX is markedly decreased in *TP53*-KO/mutant cells (Fig. 1C), and it is the only BCL-2 protein significantly decreased in all *TP53*-deficient cells compared to controls (*P* = 0.03–0.0003). Interestingly, in addition to increased MCL-1, we recently reported that AML cells with acquired resistance to VEN or to AMG176 had decreased BAX [28]. These results suggest that BAX reduction contributes to BH3 mimetic resistance in *TP53*-deficient AML cells.

### Co-targeting BCL-2 and MCL-1 overcomes BAX-dependent decreased sensitivity of *TP53*-mutant AML cells to BH3 mimetics

**Fig. 2:**
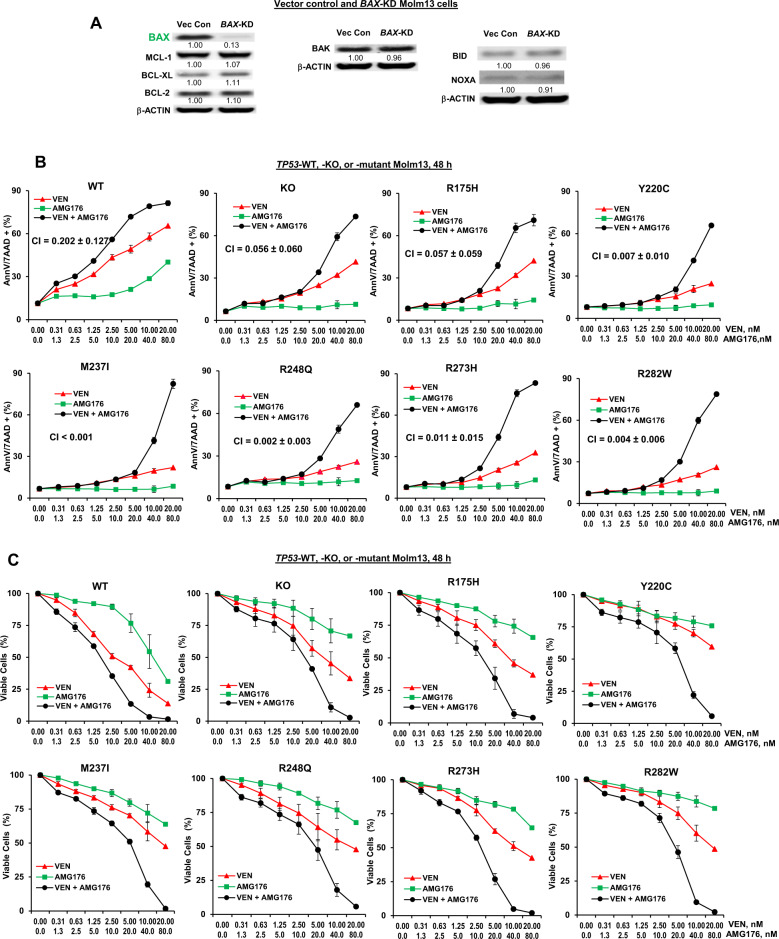
Combined inhibition of BCL-2 and MCL-1 synergistically induces cell death and decreases cell viability in *TP53*-KO and -mutant cells. **A** Western blotting of BCL-2 family proteins in vector control (Vec Con) and *BAX*-KD Molm13 cells. **B**, **C** *TP53*-WT, -KO, and -mutant Molm13 cells were treated with VEN, AMG176, or both for 48 h. Cell death (**B**) and cell viability (**C**) in the WT control cells and the *TP53*-KO and *TP53*-mutant cells were determined by flow cytometry. Experiments were performed in triplicate. Error bars represent the mean ± SEM of three independent experiments. h hour, AnnV annexin V, 7AAD 7-aminoactinomycin D.

We previously reported that AML cells with acquired resistance to VEN or AMG176 expressed increased MCL-1 and BCL-2A1 and decreased BAX levels. Although VEN or AMG176 individually were less effective in these cells, the combination of the agents was highly synergistic [28]. We reported that *BAX*-KD diminished the sensitivity of AML cells to BH3 mimetics, but that this sensitivity was restored by the combined inhibition of BCL-2 and MCL-1 [28]. To demonstrate that BAX loss itself was sufficiently responsible for the reduced sensitivity to BH3 mimetics, we examined other BCL-2 proteins in *BAX*-KD cells and found that these were largely unchanged (Fig. 2A). Because BAX decrease is common in *TP53*-deficient AML cells, we tested if co-targeting BCL-2 and MCL-1 improves the responses of *TP53*-deficient cells to BH3 mimetics. We treated isogenic *TP53*-WT, -KO, and -mutant Molm13 cells with VEN, AMG176, or both. As expected, *TP53*-KO/-mutant cells exhibited decreased sensitivity to VEN or AMG176 as compared to WT controls, but when combined, the two BH3 mimetics were highly synergistic in inducing apoptosis (CI: <0.001 to 0.057) (Fig. 2B) and effectively decreased cell viability (Fig. 2C). Although less potent at lower doses in *TP53*-KO/-mutant cells than in controls, the combination at still very low concentrations (20 nM VEN/80 nM AMG176 and even 10 nM VEN/40 nM AMG176), which were essentially ineffective as single agents in *TP53*-deficient cells, were able to effectively induce apoptosis and reduce absolute cell numbers, reflected in the exceptionally low CI values, regardless of *TP53* mutational status.

Decreased BAX levels and synergism of VEN and AMG176 combination were also found in *TP53-*R248W Molm13 cells and *TP53-*KD OCI-AML3 cells (Supplemental Fig. 3A, B). The combination was also synergistic in other *TP53*-mutant leukemia cell lines (Supplemental Fig. 3C).

We found that in addition to increased MCL-1, MV4-11 cells with acquired resistance to VEN/AMG176 combination had undetectable BAX levels (Supplemental Fig. 4), further supporting the critical role of BAX in BH3 mimetic activity.

### Co-targeting of BCL-2 and MCL-1 activates BAX and BAK and promotes cytochrome *C* release in *TP53*-mutant AML

**Fig. 3:**
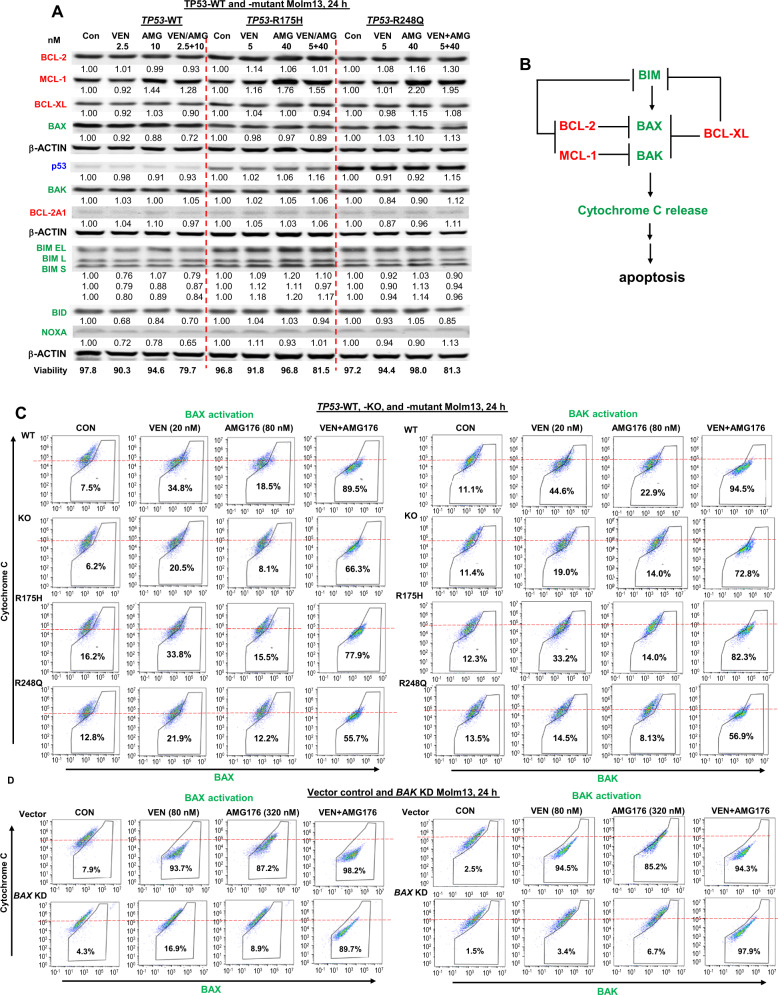
The role of BCL-2 family members in BH3 mimetic–induced apoptosis. **A** *TP53*-WT and -mutant (R175H and R248Q) Molm13 cells were treated with VEN, AMG176 (AMG), or both at the indicated concentrations for 24 h. Protein levels were determined by Western blot analysis. **B** Schematic of apoptosis induction requiring BAX and BAK activation, which is activated by BIM and blocked by BCL-2 and MCL-1. **C**, **D** *TP53*-WT, -KO, or -mutant Molm13 cells (**C**) and vector control or *BAX*-KD Molm13 cells (**D**) were treated with VEN, AMG176, or both at the indicated concentrations for 24 h. BAX and BAK activation and cytochrome *C* release were measured by flow cytometry. h hour.

Given that cells with a net decrease of anti-apoptotic proteins or a net increase of pro-apoptotic proteins are primed to undergo apoptosis, we determined levels of BCL-2 family proteins in *TP53*-WT and -mutant (R175H and R248Q) Molm13 cells treated with VEN, AMG176, or the combination. As expected, AMG176 increased MCL-1 levels in all cells examined (Fig. 3A). Although it effectively induced apoptosis, the combination did not further alter the levels of BCL-2 proteins compared to those changed by each agent alone in control or *TP53-*mutant cells. Neither agent alone, nor the combination, activated p53 in *TP53*-WT controls, in agreement with the lack of increase of BAX or NOXA.

BCL-2 family proteins cooperatively regulate apoptosis through protein-protein interaction (Fig. 3B). Using BH3 mimetic insensitive OCI-AML3 cells, we previously showed that BCL-2 inhibition decreased BCL-2–BIM and BCL-2–BAX interactions but increased MCL-1–BIM, BCL-XL–BAX, and BCL-XL–BAK interactions; while MCL-1 inhibition decreased MCL-1–BIM interactions but increased BCL-2–BIM and MCL-1–BAK interactions. Only when both were inhibited, BIM, BAX, and BAK were optimally freed from anti-apoptotic BCL-2 proteins, leading to apoptosis induction [27], supporting the notion that in BH3 mimetic less sensitive cells, modulation of multiple BCL-2 proteins is required to effectively activate BAX/BAK and induce cell death.

We treated *TP53*-WT, *-*KO, and -mutant Molm13 cells with VEN, AMG176, or both and determined the levels of activated BAX, activated BAK, and cytochrome *C* by flow cytometry. VEN or AMG176 had a limited effect on BAX or BAK activation and cytochrome *C* release in *TP53*-deficient cells as compared to WT controls, whereas the combination effectively activated BAK and BAK and induced cytochrome *C* release (Fig. 3C). Next, we treated control and *BAX*-KD Molm13 cells. Although *BAX*-KD did not alter the levels of BAK or other BCL-2 proteins (Fig. 2A), BAX and BAK activation and cytochrome *C* release were largely diminished in *BAX*-KD cells treated with VEN or AMG176. However, the combination treatment greatly induced BAX/BAK activation and cytochrome *C* release (Fig. 3D), supporting the highly synergistic effect of the combination in *TP53*-deficient AML cells based on this mechanism.

### BH3 mimetic–driven stress response and cell death patterns are independent of *TP53* mutation status

**Fig. 4:**
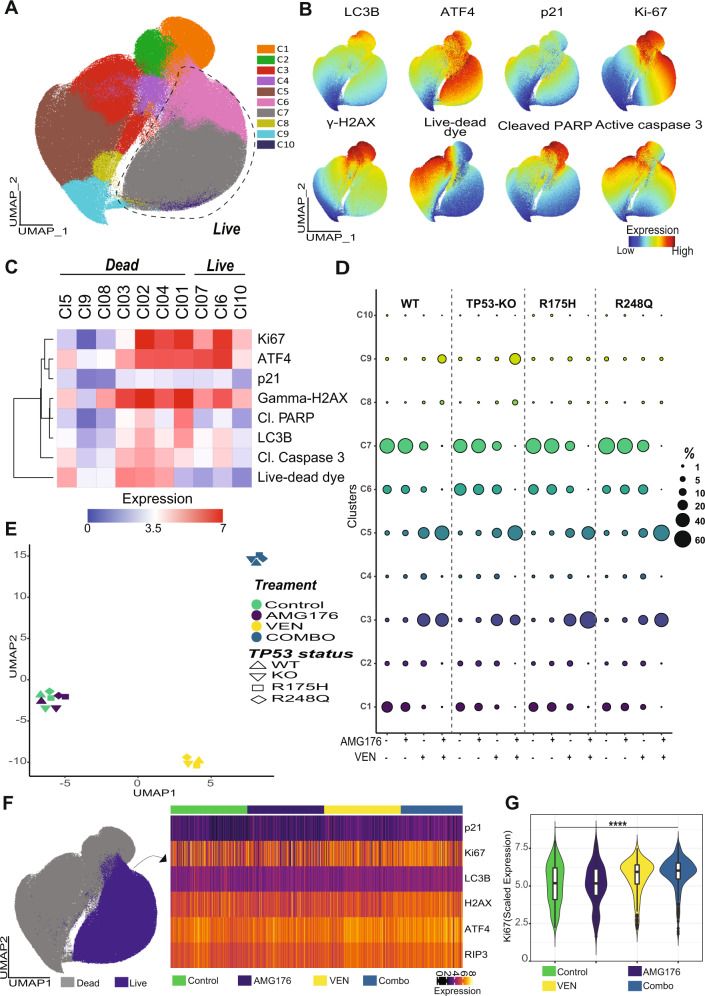
The combination of VEN and AMG176 induces similar cell death and stress patterns in *TP53*-WT, -KO, and -mutant AML cells. **A** *TP53*-WT, -KO, and -mutant (R175H and R248Q) Molm13 cells were treated with DMSO control, VEN, AMG176, or VEN plus AMG176, stained with an array of antibodies, and subjected to flow cytometry. Cells were then subjected to UMAP dimension reduction and projected on two-dimensional plots. The FlowSOM algorithm was utilized for automated cell population identification; the colors indicate FlowSOM clusters. **B** The UMAP plots for the indicated markers; the colors indicate marker expression intensity. **C** Heatmap of the arcsine-transformed expression intensities of the markers used for dimension reduction and clustering across the FlowSOM clusters shown in panel **B**. Live and dead cell clusters were identified based on the marker expression patterns shown in panel **B**. **D** Bubble plot of the frequencies of the FlowSOM clusters shown in panel **A** across *TP53*-WT, -KO, and -mutant (R175H and R248Q) Molm13 cells treated with control, VEN, AMG176, or VEN plus AMG176. **E** UMAP plot of the differential responses to treatment with control, VEN, AMG176, or VEN plus AMG176. The FlowSOM cluster frequencies shown in panel **C** were used for dimension reduction. **F** UMAP map of live cells (purple) and dead cells (gray) and heatmap of the expressions of the selected markers. **G** Violin plot of scaled Ki-67 signal intensities of *TP53*-WT, KO, and mutant leukemia cells and cells exposed to VEN, AMG176, or VEN plus AMG176 for 48 h. Live cells were selected for plotting and 200 cells (50 cells each of *TP53*-WT, KO, R175H, and R248Q) from each condition (control, AMG176, VEN, and combo) were shown. Cl cleaved, combo combination.

Because combined BCL-2 and MCL-1 inhibition can effectively activate BAX- and BAK-mediated apoptotic pathways and trigger p53-independent cell death, we reasoned that the combination would elicit similar stress responses and cell death modes irrespective of *TP53* status. Therefore, we designed a multiparametric flow cytometry panel to concomitantly assess cell death modes (apoptosis, necroptosis, and parthanatos) and stress responses (DNA damage response, proliferation, cell cycle, autophagy, and endoplasmic reticulum [ER] stress response) utilizing dimension reduction and unsupervised clustering to gain a broad overview of the treatment-specific proteomic landscape and assess whether VEN, AMG176, or co-treatment elicited overlapping or distinct proteomic shifts across leukemia cells with different *TP53* statuses. *TP53*-WT, -KO, and -mutant (R175H and R248Q) Molm13 cells were treated with VEN, AMG176, or both. We generated a concatenated data matrix that included data points from 16 different conditions (four different types of Molm13 cells treated with DMSO, VEN, AMG176, or both) and subjected the data matrix to UMAP dimension reduction and FlowSOM clustering to partition the proteomic data into distinct subpopulations (Fig. 4A and Supplemental Fig. 5). Assessment of marker expression across the overall proteomic landscape identified live and dead cells and revealed their expression patterns (Fig. 4B, C). We identified only three live-cell subpopulations. The relatively low diversity in the live-cell compartment suggests that BH3 mimetics did not induce a divergent stress response characterized by the emergence of distinct subpopulations with distinct stress patterns. Indeed, the overall proteomic architectures of leukemia cells exposed to drug combinations overlapped. We observed that VEN, AMG176, or co-treatment did not drive the emergence of distinct subsets that were not present in controls (Supplemental Fig. 5). Thus, we concluded that BH3 mimetics do not induce autophagy, ER stress, or a DNA damage response nor alter the proliferation status of leukemia cells. A lack of DNA damage and stable RIP3 expression in surviving cells ruled out parthanatos and necroptosis [42] as mechanisms of cell death upon treatment with BH3 mimetics (Fig. 4B). Assessment of the dead cell compartment revealed distinct, hierarchically organized layers of cell death (Fig. 4A, B) and caspase 3 activation and cleaved-PARP, indicating that apoptosis is the main mechanism of cell death induced by BH3 mimetics [41].

To assess whether *TP53* status alters cell death patterns and stress responses in an unsupervised manner, we quantified the fractions of leukemia cell subpopulations with different *TP53* backgrounds treated with VEN, AMG176, or both (Fig. 4D) and performed clustering analysis using the frequencies of subpopulations detected across the overall leukemia landscape to delineate the direction of the treatment response and identify the similarity of cluster composition and thus response patterns among the isogenic cells with different *TP53* statuses. We found that *TP53*-WT, -KO, and -mutant cells treated with the same drug regimen were positioned in proximity on high-dimensional space and clustered together (Fig. 4E), indicating that *TP53* status does not affect BH3 mimetic–driven stress response and cell death patterns. AMG176 alone did not induce significant cell death or a stress response; therefore, AMG176-treated cells clustered together with controls. The clustering was mainly driven by treatments, rather than *TP53* status. Leukemia cells treated with VEN alone, although less effective, were clustered together in close proximity with VEN plus AMG176-treated cells (Fig. 4E). These findings indicate that leukemia cells from distinct *TP53* backgrounds have an overlapping overall response landscape following treatment with BH3 mimetics and thus provide a rationale for using BH3 mimetics targeting both BCL-2 and MCL-1 to effectively treat *TP53*-deficient and -mutant leukemia cells.

Lastly, we selected the live cells and assessed their proteomic profiles across different conditions (Fig. 4F and Supplemental Fig. 5) to gain insights into the survival mechanisms. We observed that *TP53*-WT, -KO, or -mutant Molm13 cells with high levels of autophagy and ER stress preferentially survived after treatment with BH3 mimetics, particularly VEN alone and VEN plus AMG176 (Fig. 4F). In general, proliferating leukemia cells are more susceptible to chemotherapeutic agents or a variety of targeted therapies, but we observed that proliferating cells were preferentially enriched after treatment with BH3 mimetics, particularly VEN and VEN/AMG176 combination (Fig. 4F, G). Results suggest that proliferating cells could be less sensitive to individual BH3 mimetics, particularly to VEN (Fig. 4F). However, VEN/AMG176 combination substantially decreased the fractions of proliferating cells that would survive with VEN alone. This notion could be exploited by modulating the proliferation status of leukemia cells to render them more susceptible to BH3 mimetics.

### Combined BCL-2 and MCL-1 inhibition is effective against *TP53*-mutant AML cells and stem/progenitor cells

**Fig. 5:**
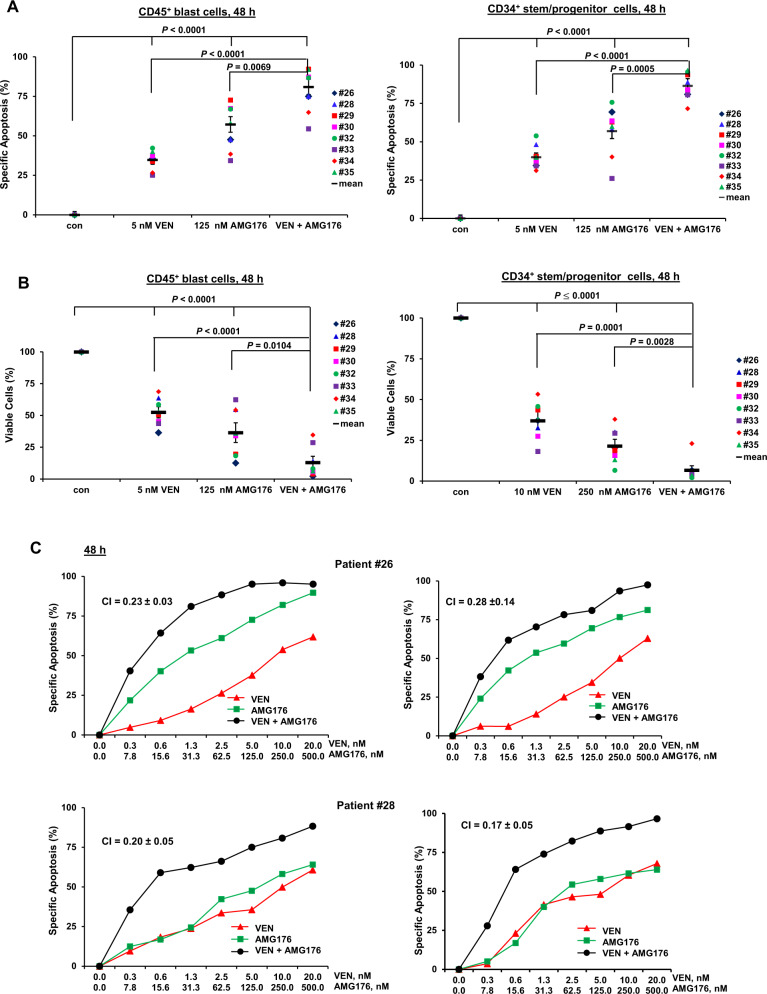
The combined inhibition of BCL-2 and MCL-1 is significantly more effective in AML cells and AML stem/progenitor cells from patients with *TP53* mutations. **A**, **B** AML patient samples (*n* = 8) with *TP53* mutations were treated with VEN, AMG176, or both for 48 h. Apoptosis (**A**) and viable cells (**B**) were determined by flow cytometry. h hour. Error bars represent mean ± SEM of data from individual patients. **C** AML patient samples (*n* = 8) with *TP53* mutations were treated with various concentrations of VEN, AMG176, or both for 48 h. Apoptosis and the CI values from two representative patient samples (#26, #28) are shown. Cell death is expressed as specific apoptosis and viable cells are expressed as % compared to the control of 100%. con control.

To validate the potential clinical relevance of targeting BCL-2 and MCL-1 in *TP53*-mutant AML mimicking a microenvironment setting in vitro, we co-cultured cells from eight AML patients with *TP53* mutations (6/8 had co-mutations, 5/8 were resistant to VEN-based therapy) (Table 1) with MSCs and treated them with VEN, AMG176, or both. In both leukemia blasts and stem/progenitor cells, the combination of VEN (5 nM) and AMG176 (125 nM) induced significantly more cell death (Fig. 5A) and reduced cell viability (Fig. 5B) compared with either agent alone. The combination of synergistically induced cell death in both CD45^+^ leukemia blasts and CD34^+^ stem/progenitor cells (Fig. 5C, patients #26 and #28). The CI values of all eight patient samples in leukemia blasts (0.04 ± 0.04 to 0.34 ± 0.10) and stem/progenitor cells (0.07 ± 0.08 to 0.28 ± 0.14) are shown in Supplemental Table 1. Data indicate that the combinatorial therapy of BCL-2 and MCL-1 inhibition is highly effective even in the presence of MSCs.

### Combined inhibition of BCL-2 and MCL-1 is highly effective in vivo in a *TP53*-WT/*TP53-*R248W Molm13 xenograft model

**Fig. 6:**
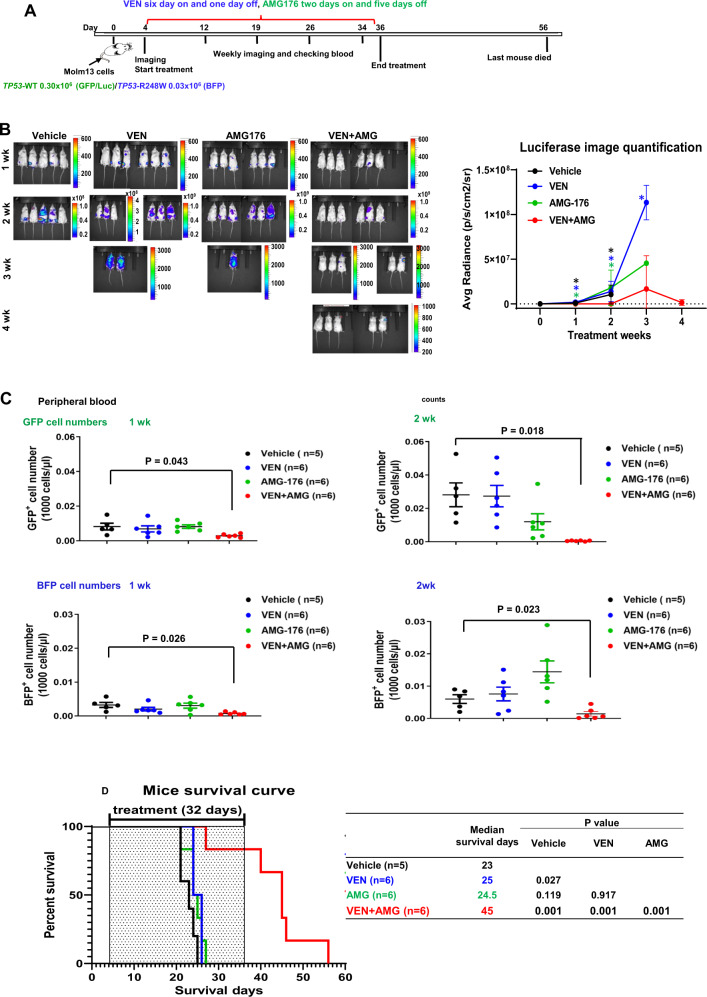
The combined inhibition of BCL-2 and MCL-1 exhibits stronger in vivo anti-leukemia activity in a xenograft mouse model. **A** Experimental scheme. **B** Bioluminescence imaging and quantification. **C** Circulating numbers of GFP^+^ and BFP^+^ cells after week 1 (left) and week 2 (right) of treatment as determined by flow cytometry. **D** Survival. *n* = 5 or 6/group. AMG AMG176, wk week. Error bars represent the mean ± SD of data from individual mice.

To assess the therapeutic potential of co-targeting BCL-2 and MCL-1 in vivo and mimic the clinical scenario, we performed experiments using xenografts of luciferase/GFP-labeled *TP53*-WT and BFP-labeled *TP53-*R248W Molm13 cell mixtures (10:1) in NSG mice (Fig. 6A). In vivo imaging analysis showed that the combination treatment significantly decreased *TP53*-WT Molm13 compared to the control or either BH3 mimetic alone at weeks-1 and -2 (Fig. 6B). At week-3, none of the controls and only one AMG176-treated mouse was alive; the combination was more effective than VEN alone. To distinguish the effects of treatments on *TP53*-WT and -mutant leukemia cells, we measured circulating GFP (*TP53-*WT)- and BFP (*TP53-*R248W)-labeled cells by flow cytometry. VEN or AMG176 alone had minimal effects on either *TP53*-WT or *TP53-*R248W Molm13 cells, and their combination significantly reduced both cell types (Fig. 6C). There was little difference between the median survival duration of control mice (23 days) and that of AMG176-treated (24.5 days) or VEN-treated mice (25 days). The median survival duration of the combination-treated mice (45 days) was significantly longer than those of the control, VEN-, and AMG176-treated mice (*P* = 0.001 for all three comparisons) (Fig. 6D).

## Discussion

We demonstrate that the significant reduction of BAX is critical for the decreased sensitivity of *TP53*-mutant AML to individual BH3 mimetics targeting BCL-2 or MCL-1. The combined inhibition of both effectively activates BAX and synergistically decreases cell viability primarily by apoptosis induction in AML cells largely independent of *TP53* mutational status. Hence, the diminished activity of BH3 mimetics in *TP53*-defective cells can be circumvented in a p53-independent manner by co-targeting BCL-2 and MCL-1.

CRISPR genome editing analyses have demonstrated that the loss of WT *TP53* or p53-regulated apoptosis regulators, such as BAX, confers VEN resistance [18]. *BAX* mutations have been identified as a mechanism of BH3 mimetic resistance in AML [29]. *BAX* mutations have also been detected in the myeloid compartment of CLL patients with clonal hematopoiesis who were treated with VEN [46], which further supports the notion that BAX has a critical role in BH3 mimetic–induced death of myeloid leukemia cells. In the present study, we found significantly decreased expression levels of BAX in *TP53-*mutant patient samples and isogenic AML cells compared to *TP53*-WT AML cells. We also found that genetic silencing of BAX rendered AML cells resistant to BH3 mimetics [28] and that BAX is largely undetectable in AML cells with acquired resistance to both VEN and AMG176. Furthermore, *TP53*-deficiency or *BAX*-KD alone sufficiently inhibits BAX and BAK activation. Collectively, these findings show that the reduction or loss of BAX are critical for BH3 mimetic resistance and contributes to the vastly reduced activity of BH3 mimetics in *TP53*-mutant AML.

Following exposure to chemotherapeutic agents, *TP53*-mutant clones are commonly selected and frequently become dominant, conferring further resistance to other therapies [47, 48]. Although efforts have been made to target selected *TP53*-mutants [49, 50], these approaches have limited clinical applicability, as >100 *TP53* mutations have been identified in AML [25]. We here demonstrate that the combined inhibition of BCL-2 and MCL-1 circumvents the resistance in *TP53*-deleted or mutant-AML to individual BH3 mimetics and synergistically induces *TP53*-independent profound apoptosis in AML cells and AML stem/progenitor cells in vitro and in vivo. Hence, our approach has potentially broad clinical implications, beyond approaches targeting selective *TP53* mutations.

Despite effectiveness, the combined inhibition of BCL-2 and MCL-1 was less robust in *TP53*-deficient/-mutant than in *TP53-*WT AML. Achieving efficacies in *TP53*-deficient/-mutant AML that were similar to that in WT AML required higher doses of both agents. Nevertheless, xenograft-bearing mice with *TP53*-mutant AML cells under combination treatment survived almost twice as long than those treated with either agent alone using the same dose and treatment schedule as we previously described, in which a VEN/HMA resistant *TP53*-WT PDX model showed a three-fold survival extension [28]. AMG176 and VEN/AMG176 clinical trials in hematological malignancies have been initiated. Careful titration of the doses of each BH3 mimetic to maximize the combination’s efficacy and minimize toxicity will hopefully result in an effective and safe combinatorial regimen.

Besides direct inhibition, indirect targeting MCL-1 through mechanisms such as inhibition of MAPK or CDK9 could also sensitize AML cells to VEN. Given the critical role of BAX in mitochondria-regulated apoptosis, pharmacologically or genetically increasing BAX levels may improve the lethality of BH3 mimetics and benefit patients whose disease is resistant to BH3 mimetics. In fact, with an improved understanding of the structure-function relationship of BAX, researchers are developing strategies to modulate BAX activity or expression with various approaches, including small molecules and peptides [51]. All approaches that increase pro- and decrease anti-apoptotic BCL-2 have the potential to sensitize *TP53*-mutant AML cells to VEN and warrant further exploration.

## Supplementary information

Supplemental Data

## Data Availability

Materials described in the manuscript, including all relevant raw data, will be freely available to any researcher wishing to use them for non-commercial purposes, without breaching participant confidentiality.
