# Correction: Combined inhibition of BCL-2 and MCL-1 overcomes BAX deficiency-mediated resistance of *TP53*-mutant acute myeloid leukemia to individual BH3 mimetics

**DOI:** 10.1038/s41408-023-00857-z

**Published:** 2023-05-17

**Authors:** Bing Z. Carter, Po Yee Mak, Wenjing Tao, Edward Ayoub, Lauren B. Ostermann, Xuelin Huang, Sanam Loghavi, Steffen Boettcher, Yuki Nishida, Vivian Ruvolo, Paul E. Hughes, Phuong K. Morrow, Torsten Haferlach, Steven Kornblau, Muharrem Muftuoglu, Michael Andreeff

**Affiliations:** 1 Section of Molecular Hematology and Therapy, Department of Leukemia, The University of Texas MD Anderson Cancer Center, Houston, TX USA; 2 Department of Biostatistics, The University of Texas MD Anderson Cancer Center, Houston, TX USA; 3 Department of Hematopathology, The University of Texas MD Anderson Cancer Center, Houston, TX USA; 4 Department of Medical Oncology and Hematology, University Hospital Zurich and University of Zurich, Zurich, Switzerland; 5 Oncology Research, Amgen Inc, Thousand Oaks, CA USA; 6 Amgen Inc, Thousand Oaks, CA USA; 7 MLL Munich Leukemia Laboratory, Munich, Germany

**Keywords:** Cancer, Leukaemia

Correction to: *Blood Cancer Journal* 10.1038/s41408-023-00830-w, published online 24 April 2023

The following acknowledgement was missing from this article.

EA is a TRIUMPH Fellow in the CPRIT Training Program (RP210028).

The original article has been corrected.

