# Supplementary material for: Combined inhibition of BCL-2 and MCL-1 overcomes BAX deficiency-mediated resistance of TP53-mutant acute myeloid leukemia to individual BH3 mimetics
Source: Blood Cancer J. 2023 Apr 24;13(1):57. doi: 10.1038/s41408-023-00830-w (PMC10123065; doi:10.1038/s41408-023-00830-w)
Supplement: Supplementary file 1 — Supplemental Data [file 41408_2023_830_MOESM1_ESM.pdf]

Supplemental Data for

Combined Inhibition of BCL-2 and MCL-1 Overcomes BAX Deficiency-Mediated Resistance of *TP53*-mutant Acute Myeloid Leukemia to Individual BH3 Mimetics

Bing Z. Carter,[1] Po Yee Mak,[1] Wenjing Tao,[1] Edward Ayoub,[1] Lauren B. Ostermann,[1] Xuelin Huang,[2] Sanam Loghavi,[3] Steffen Boettcher,[4] Yuki Nishida,[1] Vivian Ruvolo,[1] Paul E. Hughes,[5] Phuong K. Morrow,[6] Torsten Haferlach,[7] Steven Kornblau,[1] Muharrem Muftuoglu,[1] and Michael Andreeff[1]
[1]Section of Molecular Hematology and Therapy, Department of Leukemia, [2]Department of Biostatistics, and [3]Department of Hematopathology, The University of Texas MD Anderson Cancer Center, Houston, TX; [4]Department of Medical Oncology and Hematology, University Hospital Zurich and University of Zurich, Zurich, Switzerland; [5]Oncology Research, Amgen Inc., Thousand Oaks, CA; and [6]Amgen Inc., Thousand Oaks, CA; [7]MLL Munich Leukemia Laboratory, Munich, Germany.

Supplemental Table 1. Combination index (CI) values of patient samples treated with venetoclax and AMG176 for 48 hours

	CI values	
Patient no.	CD45$^+$ cells	CD34$^+$ cells
26	0.23 ± 0.03	0.28 ± 0.14
28	0.20 ± 0.05	0.17 ± 0.05
29	0.34 ± 0.10	0.22 ± 0.08
30	0.25 ± 0.19	0.22 ± 0.12
32	0.15 ± 0.07	0.07 ± 0.08
33	0.04 ± 0.04	0.24 ± 0.16
34	0.29 ± 0.07	0.21 ± 0.07
35	0.19 ± 0.15	0.15 ± 0.03

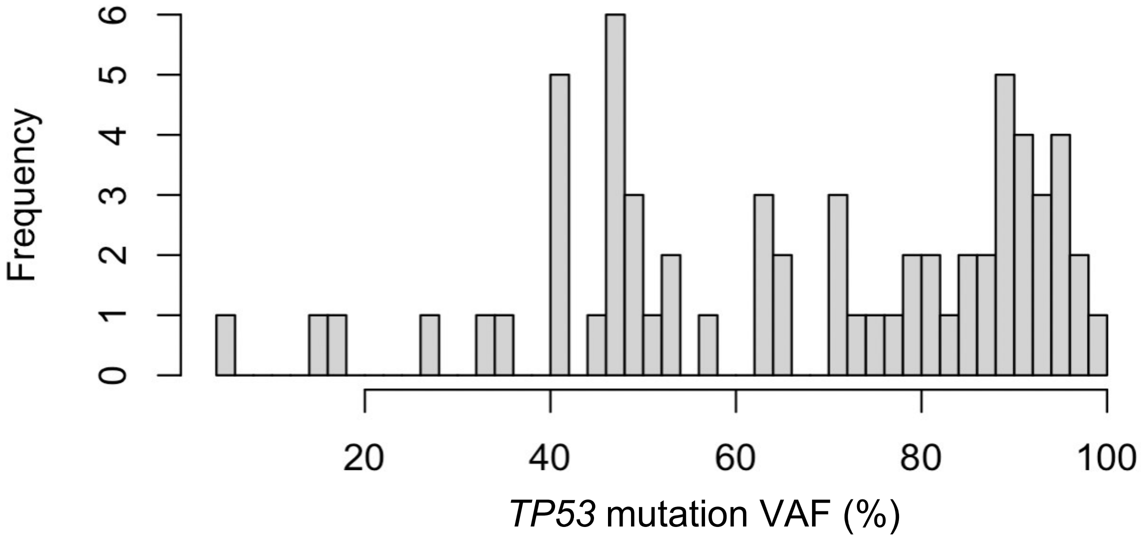

Supplemental Figure 1. VAF frequency distribution of *TP53*-mutant AML patient samples used for RNA-seq analysis.

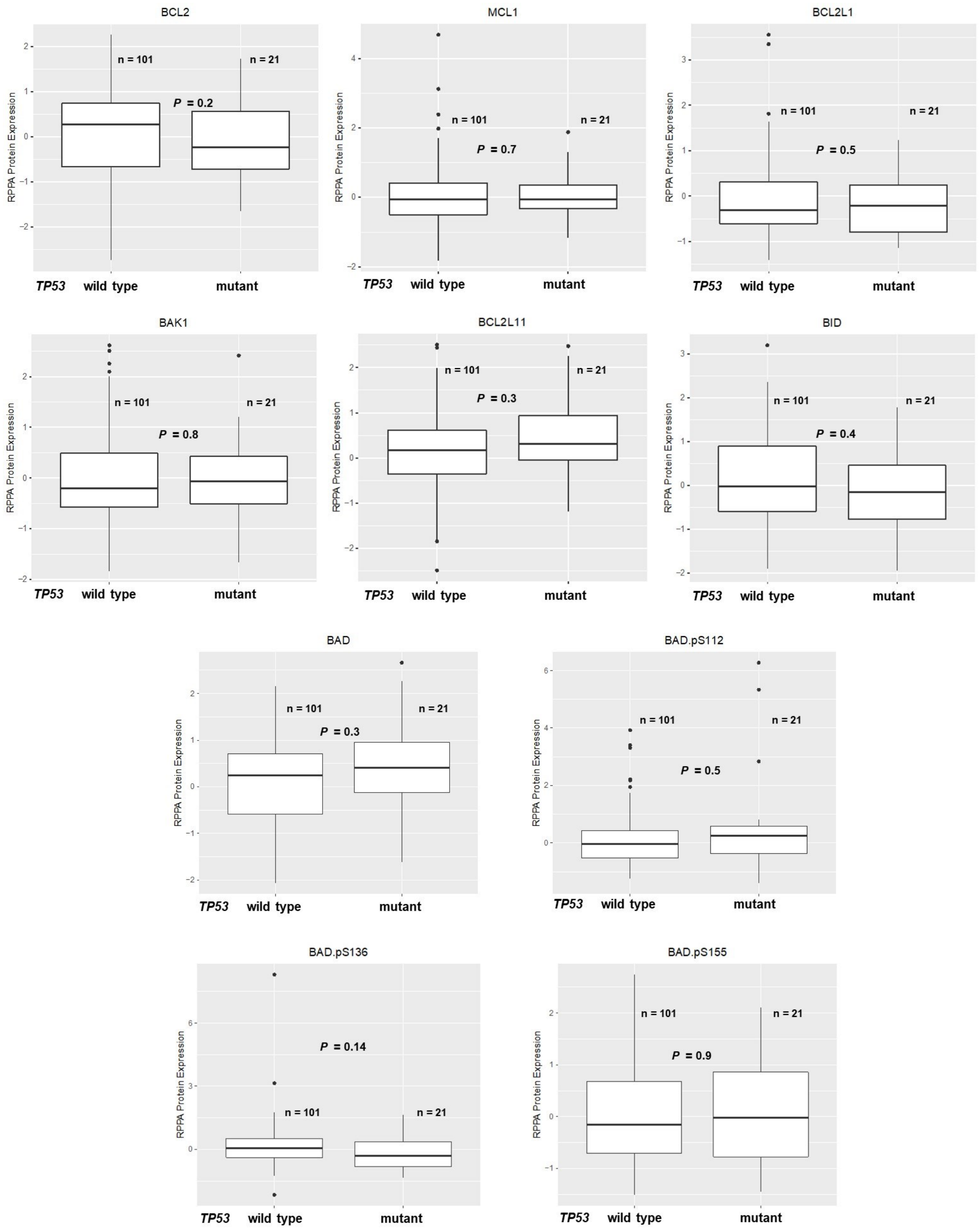

Supplemental Figure 2. RPPA analysis of BCL-2 family protein expression in patients with newly diagnosed *de novo TP53*-WT (n = 101) or *TP53*-mutant (n = 21) AML.

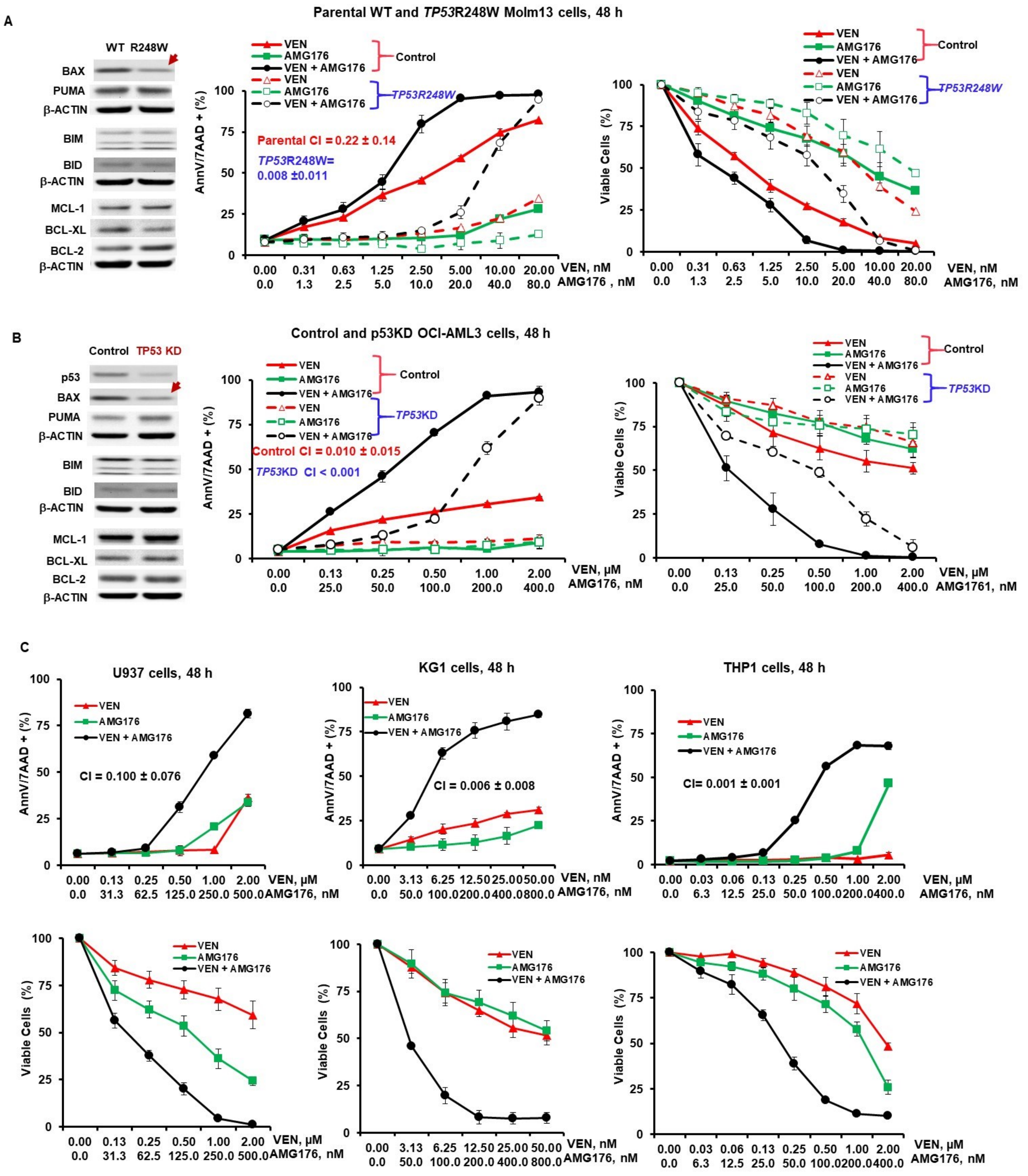

Supplemental Figure 3. The combined inhibition of BCL-2 and MCL-1 is highly synergistic against *TP53*-mutant and -KD leukemia cells. A–C. Control and *TP53*-R248W Molm13 cells (A), control and *TP53*-KD OCI-AML3 cells (B), and other *TP53*-mutant leukemia cells (C) were treated with venetoclax (VEN), AML176, or both. Cell death and viability were determined by flow cytometry. Experiments were performed in triplicates, and data are expressed as mean ± SEM.

Protein levels were determined by Western blot analysis and the arrows mark the decreased expression. AnnV, annexin V; 7AAD, 7-aminoactinomycin D.

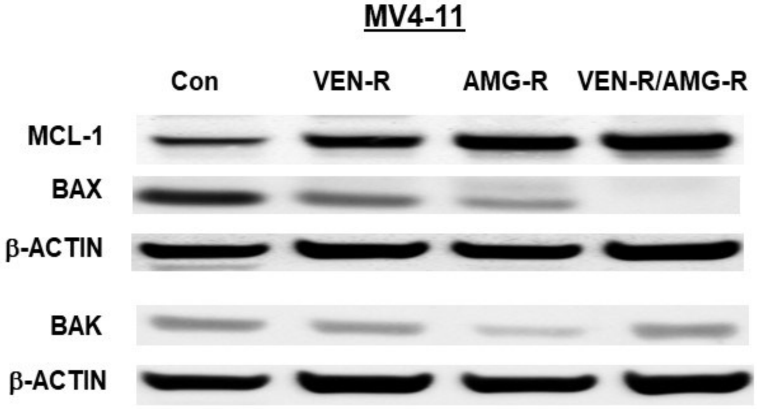

Supplemental Figure 4. Expression of BCL-2 family proteins in MV4-11 cells with acquired resistance to venetoclax (VEN-R), AMG176 (AMG-R), or both (VEN-R/AMG-R), as assessed by Western blotting. Con, control.

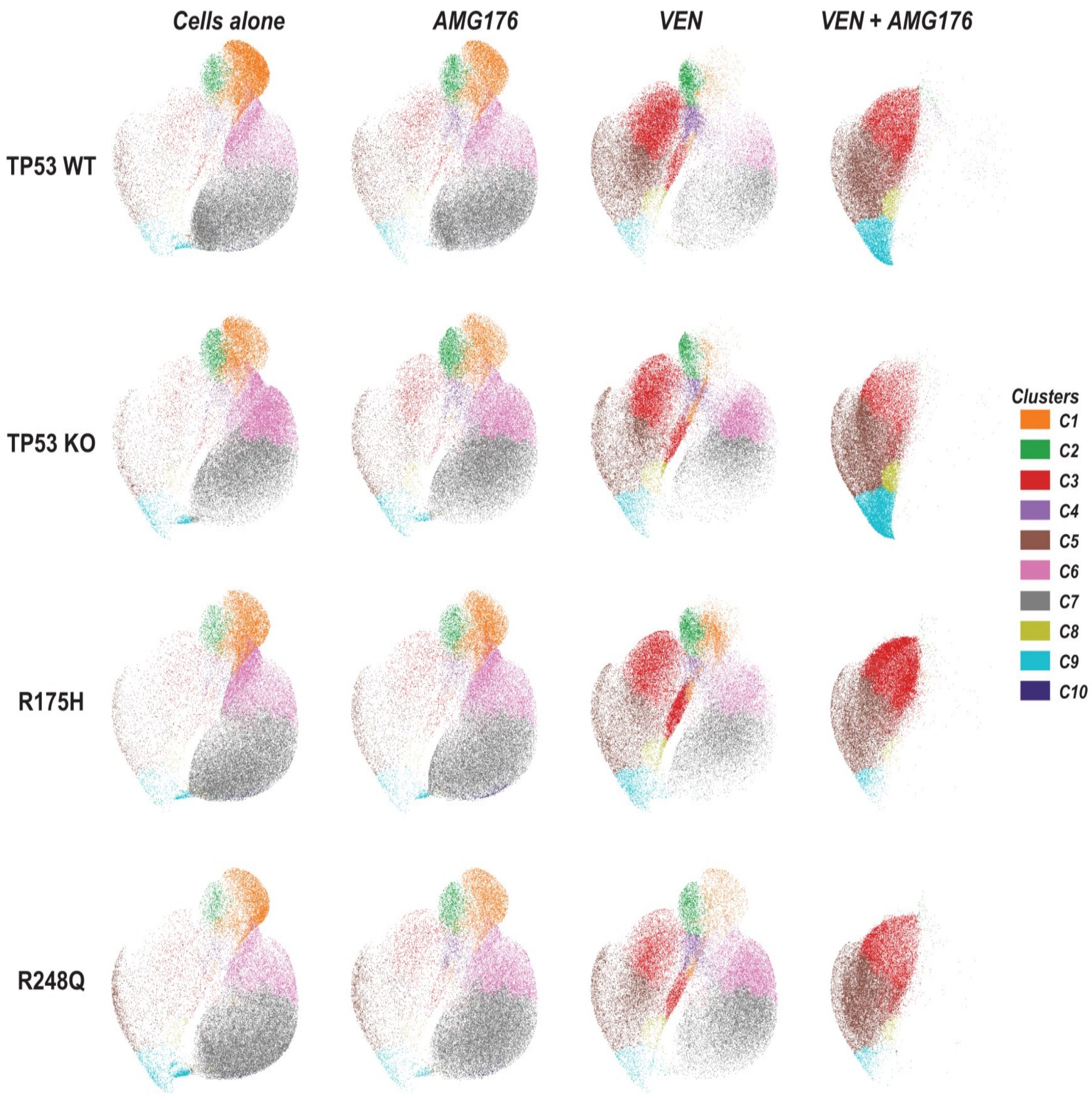

Supplemental Figure 5. The combination of venetoclax plus AMG176 induces similar cell death and stress patterns in *TP53*-WT, -deficient, and -mutant AML cells. *TP53*-WT, -KO, and -mutant (R175H and R248Q) Molm13 cells were treated with DMSO, VEN, AMG176, or the combination and stained with an array of flow cytometry antibodies. The cells were then subjected to UMAP dimension reduction and projected on 2-dimensional plots. The FlowSOM algorithm was utilized for automated cell population identification; the colors indicate different FlowSOM clusters.
